# A phase Ib/IIa clinical trial of dantrolene sodium in patients with Wolfram syndrome

**DOI:** 10.1172/jci.insight.145188

**Published:** 2021-08-09

**Authors:** Damien Abreu, Stephen I. Stone, Toni S. Pearson, Robert C. Bucelli, Ashley N. Simpson, Stacy Hurst, Cris M. Brown, Kelly Kries, Chinyere Onwumere, Hongjie Gu, James Hoekel, Lawrence Tychsen, Gregory P. Van Stavern, Neil H. White, Bess A. Marshall, Tamara Hershey, Fumihiko Urano

**Affiliations:** 1 Division of Endocrinology, Metabolism, and Lipid Research, Department of Medicine,; 2 Medical Scientist Training Program,; 3 Division of Endocrinology and Diabetes, Department of Pediatrics,; 4 Department of Neurology,; 5 Center for Clinical Studies,; 6 Division of Biostatistics,; 7 Department of Ophthalmology & Visual Sciences,; 8 Departments of Psychiatry and Radiology, and; 9 Department of Pathology & Immunology, Washington University School of Medicine, St. Louis, Missouri, USA.

**Keywords:** Endocrinology, Genetics, Cell stress, Diabetes, Genetic diseases

## Abstract

**BACKGROUND:**

Wolfram syndrome is a rare ER disorder characterized by insulin-dependent diabetes mellitus, optic nerve atrophy, and progressive neurodegeneration. Although there is no treatment for Wolfram syndrome, preclinical studies in cell and rodent models suggest that therapeutic strategies targeting ER calcium homeostasis, including dantrolene sodium, may be beneficial.

**METHODS:**

Based on results from preclinical studies on dantrolene sodium and ongoing longitudinal studies, we assembled what we believe is the first-ever clinical trial in pediatric and adult Wolfram syndrome patients with an open-label phase Ib/IIa trial design. The primary objective was to assess the safety and tolerability of dantrolene sodium in adult and pediatric Wolfram syndrome patients. Secondary objectives were to evaluate the efficacy of dantrolene sodium on residual pancreatic β cell functions, visual acuity, quality-of-life measures related to vision, and neurological functions.

**RESULTS:**

Dantrolene sodium was well tolerated by Wolfram syndrome patients. Overall, β cell functions were not significantly improved, but there was a significant correlation between baseline β cell functions and change in β cell responsiveness (*R*^2^, *P* = 0.004) after 6-month dantrolene therapy. Visual acuity and neurological functions were not improved by 6-month dantrolene sodium. Markers of inflammatory cytokines and oxidative stress, such as IFN-γ, IL-1β, TNF-α, and isoprostane, were elevated in subjects.

**CONCLUSION:**

This study justifies further investigation into using dantrolene sodium and other small molecules targeting the ER for treatment of Wolfram syndrome.

**TRIAL REGISTRATION:**

ClinicalTrials.gov identifier NCT02829268

**FUNDING:**

NIH/National Institute of Diabetes and Digestive and Kidney Diseases (NIDDK) (DK112921, DK113487, DK020579), NIH/National Center for Advancing Translational Sciences (NCATS) (TR002065, TR000448), NIH training grant (F30DK111070), Silberman Fund, Ellie White Foundation, Snow Foundation, Unravel Wolfram Syndrome Fund, Stowe Fund, Eye Hope Foundation, Feiock Fund, Washington University Institute of Clinical and Translational Sciences grant UL1TR002345 from NIH/NCATS, Bursky Center for Human Immunology & Immunotherapy Programs.

## Introduction

Wolfram syndrome is an ultrarare, progressive neurodegenerative disorder characterized by juvenile-onset insulin-requiring diabetes mellitus and optic nerve atrophy (1, 2). Other clinical manifestations of Wolfram syndrome include diabetes insipidus, deafness, neurogenic bladder, and ataxia. Most individuals with Wolfram syndrome have a shortened life span due to severe neurological disabilities caused by brainstem and cerebellar atrophy (3). There has yet to be a treatment devised that has been shown to provide a cure or slow the insidious progression of this disease. As a result, patients with Wolfram syndrome are currently only offered therapies aimed at treating each aspect of the disease individually.

Since the discovery of Wolfram syndrome 1 gene (*WFS1*) as the causative locus for most cases of Wolfram syndrome, research efforts have sought to understand the underlying etiology of this disorder. Our current understanding is that Wolfram syndrome is a prototype of ER disease in humans (4). WFS1 is a multipass ER transmembrane protein with an established role in the negative regulation of ER stress and the maintenance of cellular calcium homeostasis (5, 6). While the molecular details of WFS1 function require further study, it is clear that pancreatic β cells and neurons are particularly affected by, and perhaps especially sensitive to, disease-causing *WFS1* genetic variants. Indeed, previous reports from our lab identified calcium dyshomeostasis as a key mechanism underlying pancreatic β cell and neuronal cell death in the context of WFS1 depletion (7–9). These preclinical studies led to identifying dantrolene sodium as a potential therapeutic candidate for restoring ER calcium homeostasis and mitigating the progression of Wolfram syndrome.

Dantrolene sodium is a hydantoin derivative skeletal muscle relaxant whose mechanism of action revolves around the inhibition of ryanodine receptors (RyRs) on the ER (10–12). Although the mechanism of action of dantrolene remains unclear, its effects are well-documented. Dantrolene inhibits ER calcium efflux through RyRs, thereby reducing cytosolic calcium and preserving ER calcium (13). The primary indication for dantrolene is in the treatment of malignant hyperthermia, which can be an adverse reaction to general anesthesia and is FDA approved for use in both adults and children. Dantrolene has also been used off-label for the treatments of spasticity disorders and cerebral vasospasm (14, 15). Interestingly, recent studies have proposed a potential role for dantrolene sodium as a treatment for neurodegenerative disorders, such as Huntington’s disease (16), spinocerebellar ataxia (17, 18), and Alzheimer’s disease (19, 20), where ER calcium may play a pivotal role in disease pathogenesis.

We showed positive preclinical results in mouse and induced pluripotent stem cell (iPSC) models of Wolfram syndrome treated with dantrolene sodium before (8). Based on those results, our group put together what we believe is the first-ever clinical trial in subjects with Wolfram syndrome. Our research team was particularly sensitive to the unique challenges of performing a clinical trial for a disease as rare as Wolfram syndrome (21). These challenges include the small numbers of subjects available to study and the vast heterogeneity of symptoms exhibited by patients with Wolfram syndrome (22). Multiple stakeholders were involved in the design of this clinical trial, including I-TRAK (a natural history study of neurodegeneration in early Wolfram syndrome, ClinicalTrials.gov NCT03951298) and various Wolfram syndrome patient/parent advocacy groups because they would be likely sources for recruitment. After these collaborations, an open-label phase Ib/IIa trial design was chosen. The primary endpoint of the study was to assess the safety and tolerability of dantrolene sodium in adult and pediatric subjects with Wolfram syndrome. Secondary objectives were to assess the effect of dantrolene sodium on residual pancreatic β cell function, visual acuity, neurological function, and quality-of-life measures.

## Results

### Trial population

**Figure 1:**
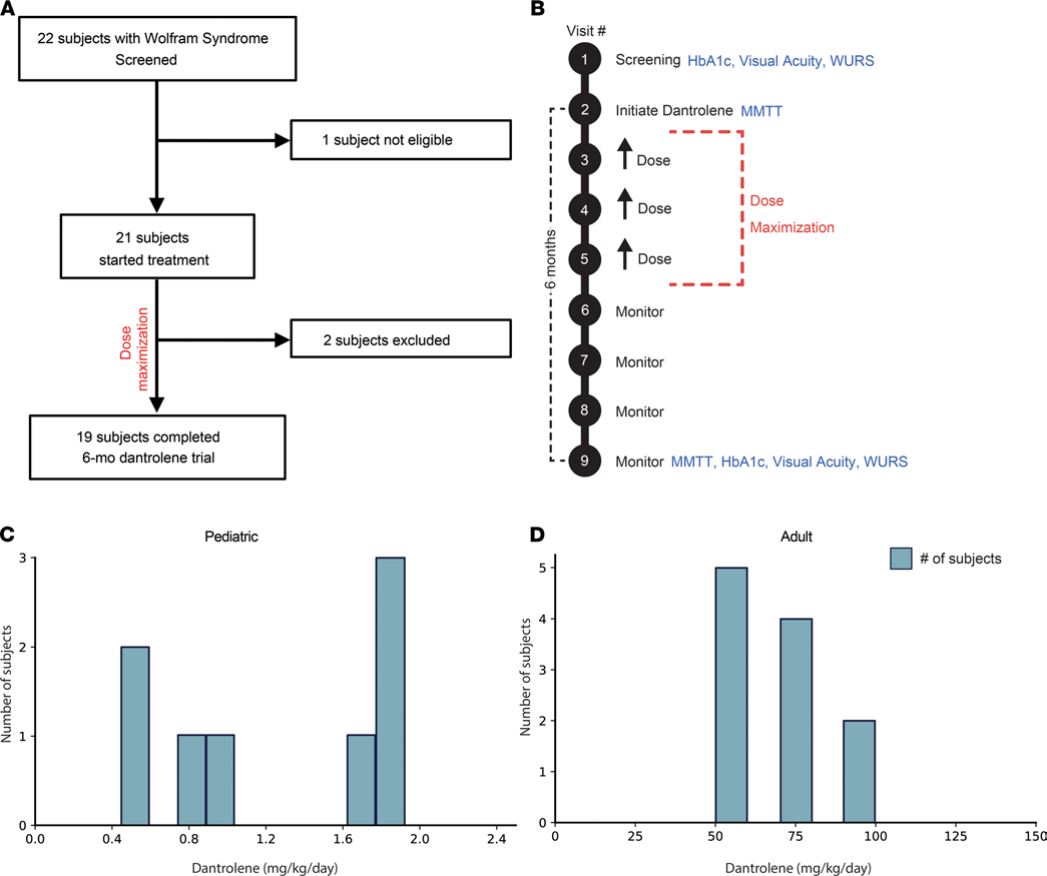
Trial design, enrollment, and retention. (**A**) Enrollment and retention diagram for the subjects enrolled in the study. (**B**) Schematic of 6-month study. Each study visit is noted by a black circle. Study procedures for secondary endpoints are noted in blue. The dose maximization period for dantrolene sodium is noted by the red dashed lines. HbA1c, hemoglobin A1c; MMTT, mixed meal tolerance test; WURS, Wolfram Unified Rating Scale. (**C**) Histogram demonstrating distribution of final tolerated dantrolene doses in pediatric subjects at the end of the study. For pediatric subjects this is expressed as mg/kg/d. (**D**) Histogram demonstrating distribution of final tolerated dantrolene doses in adult subjects at the end of the study. For adult subjects this is expressed as mg/d. For both histograms the blue bars represent numbers of subjects taking a dose.

A total of 22 subjects (6–32 years old), with a genetically confirmed diagnosis of Wolfram syndrome, were screened for enrollment in this study (Figure 1, A and B). Of this group, 21 qualified for baseline laboratory and quality-of-life assessments in order to begin the run-in regimen of oral dantrolene parallel to ongoing maintenance medications. Two subjects (11% of qualified population) had to be excluded before the 6-month assessment of study outcome measures on dantrolene treatment due to loss to follow-up or personal reasons. The baseline demographics and clinical characteristics of the 19 subjects who completed the trial are shown in Table 1. The subjects or their parents made the classifications of demographics. Subject-specific *WFS1* mutations and clinical data are summarized in Supplemental Table 1; supplemental material available online with this article; https://doi.org/10.1172/jci.insight.145188DS1 The baseline therapies the subjects took are shown in Supplemental Table 2. At enrollment 100% of subjects carried a diagnosis of diabetes mellitus. However, only 63% of pediatric subjects and 100% of adult subjects carried a diagnosis of optic atrophy. This pattern is consistent with the documented natural history of Wolfram syndrome, where juvenile-onset diabetes mellitus typically manifests within the first decade, followed by optic atrophy in the second decade of life (3, 23, 24).

### Safety outcomes

Dantrolene was well tolerated among pediatric subjects at a final daily dose between 0.5 mg/kg and 2.0 mg/kg, with a maximum daily dose of 100 mg. The mean final daily dose in the pediatric subjects was 1.25 mg/kg/d (Figure 1C). Adult subjects tolerated dantrolene well between 50 mg and 100 mg daily. Respectively, 5, 4, and 2 subjects tolerated 50, 75, and 100 mg of dantrolene. This resulted in a mean dose of 68.2 mg daily (Figure 1D). These dosing ranges closely approximated therapeutic ranges for dantrolene when used to treat spasticity (25).

Adverse events were stratified into 3 categories based on their likelihood of being attributed to the study drug. These categories included adverse events attributed directly to dantrolene, events known to occur commonly in patients with Wolfram syndrome, and nonspecific events not easily attributed to dantrolene or Wolfram syndrome. The most common adverse dantrolene-related events observed in pediatric and adult subjects were mild fatigue and diarrhea. The most common Wolfram syndrome–related events were mild hypoglycemia and headaches. These symptoms affected at least 25% of the total study population (Table 2). Hepatotoxicity and weakness, the most serious known side effects of dantrolene, were not very prevalent in our study population. Elevated liver enzymes were observed in 2 subjects (11% of total population), and weakness was self-reported by 4 subjects (21% of total population). Quantitative assessments of strength prior to and at each subsequent trial visit after dantrolene administration showed no significant loss in grip strength during 6 months of dantrolene treatment (Supplemental Figure 1). No clinically significant changes in laboratory measures or in findings from physical examinations were noted during enrollment in this study. No significant ECG changes were observed in subjects during the run-in period or thereafter. Additionally, no subject discontinued the trial regimen due to adverse effects.

### Secondary outcomes

#### Markers of β cell function.

To assess the effect of dantrolene on glycemia and remaining β cell function, HbA1c and 30-minute mixed meal stimulated C-peptide were monitored at baseline and after 6 months of dantrolene treatment (Figure 1B). Mean HbA1c across all subjects remained stable between dantrolene initiation and after 6 months of treatment (7.4% ± 0.2%, *P* value 0.63) (Table 3). Subgroup analyses of adult and pediatric subjects also demonstrated no significant change in HbA1c (7.4% ± 0.2%) (Table 4). Mean fasting C-peptide levels of the total study cohort also remained stable during this period (0.27 ± 0.07 ng/mL at 6 months of treatment compared to 0.27 ± 0.06 ng/mL at baseline, *P* value 0.95) (Table 3). At the conclusion of the study, mean stimulated C-peptide levels were not significantly higher compared to the pretreatment baseline (0.64 ± 0.14 ng/mL after 6 months of treatment compared to 0.52 ± 0.10 ng/mL at baseline, *P* value 0.14) (Table 3). Supplemental Figure 2 demonstrates subject-specific change in fasting and stimulated C-peptide over the 6-month study period. When looking at all subjects, ΔC-peptide (the change in C-peptide between 0 and 30 minutes) was not significantly increased. Mean ΔC-peptide was 0.37 ± 0.07 ng/mL after 6 months of treatment, compared to 0.25 ± 0.04 ng/mL at baseline (*P* value 0.18) (Table 3).

Additional markers of β cell function were assessed, including measuring proinsulin along with C-peptide during the mixed meal tolerance testing. Insulinogenic index (26) and AUC C-peptide/AUC glucose (27) were calculated for each study subject. No significant differences were found in any of these categories (Tables 3–5).

#### Subgroup analyses.

**Figure 2:**
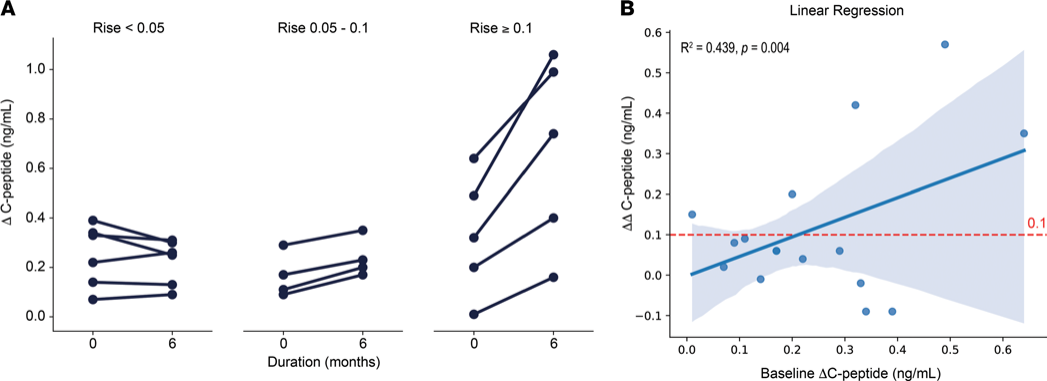
Subgroup analysis to determine responders versus nonresponders. (**A**) ΔC-peptide was plotted between all subjects. Then the change in ΔC-peptide (ΔΔC-peptide) was calculated for each subject over the course of the study. Subjects were stratified based on a ΔΔC-peptide < 0.05, 0.05–0.1, and ≥ 0.1 ng/mL, respectively. (**B**) Linear regression analysis demonstrates a significant positive relationship between baseline ΔC-peptide and ΔΔC-peptide. Statistical significance was determined by the ordinary least squares method.

**Figure 3:**
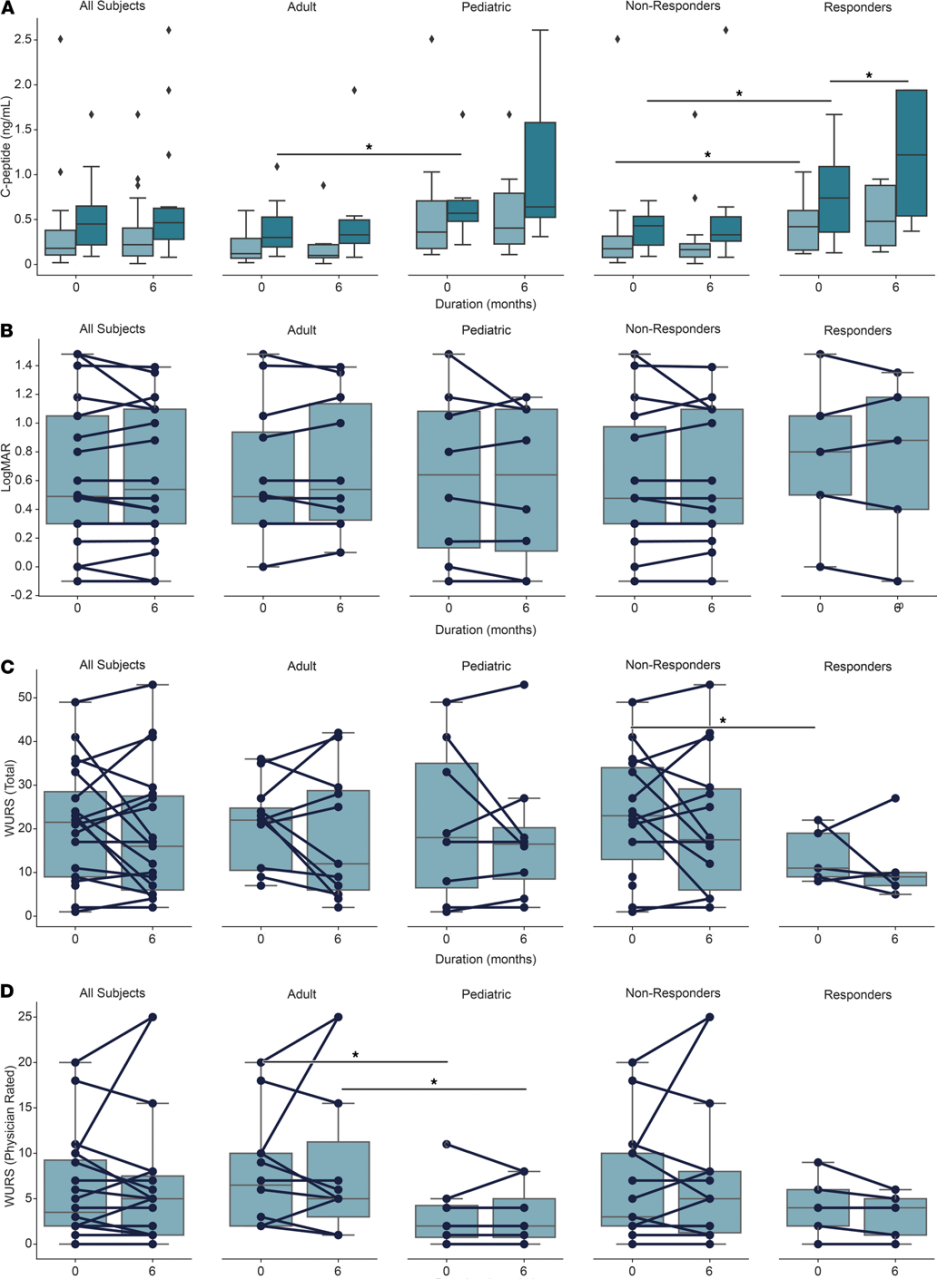
Secondary study endpoints. (**A**) C-peptide during an MMTT. Light boxes represent fasting results, while dark boxes represent 30-minute (stimulated) values. (**B**) LogMAR (a measure of visual acuity). Lower score correlates to more accurate vision. (**C**) WURS score. (**D**) Physician-rated subsection of the WURS. Higher WURS scores represent more severe disease. All study subjects are broken down into adult and pediatric subgroups. Responders are differentiated from nonresponders by having a change in ΔC-peptide (ΔΔC-peptide) ≥ 0.1 ng/mL over the study period. The box shows the quartiles of the data set while the whiskers extend to show the rest of the distribution. Paired analyses among the same group (i.e., adult or pediatric) were performed using the Wilcoxon signed-rank test. The Mann-Whitney *U* test was used when comparing between groups (adult vs. pediatric subjects or nonresponders vs. responders).

Additional subgroup analysis was performed in the study population to determine if there was a subset of subjects who had the most beneficial response to dantrolene. Our hypothesis was that subjects who possessed the greatest degree of β cell function at baseline would have the greatest glycemic benefit from dantrolene. Therefore, we examined the change in ΔC-peptide (ΔΔC-peptide) over the course of the study to approximate changes in β cell responsiveness. ΔΔC-peptide was calculated by subtracting the ΔC-peptide at baseline from the ΔC-peptide at 6 months. To test our hypothesis, we divided the subjects based on increasing cutoffs of ΔΔC-peptide (<0.05, 0.05–0.1, and >0.1 ng/mL). We noted that 5 subjects had a ΔΔC-peptide ≥ 0.1 ng/mL, 4 subjects had a ΔΔC-peptide 0.05–0.1 ng/mL, and 6 subjects had a ΔΔC-peptide < 0.05 ng/mL. The remaining subjects were missing data necessary for calculation of ΔΔC-peptide because we could not perform the MMTT due to their high fasting blood glucose levels (Figure 2A and Figure 3A). Review of these data suggested that our hypothesis was correct, as subjects with higher ΔC-peptide to begin with tended to have a higher ΔC-peptide at 6 months and higher slope (ΔΔC-peptide). To further test this relationship, we performed linear regression analysis, demonstrating a statistically significant (*R*^2^ 0.439, *P* value 0.004) positive relationship between baseline ΔC-peptide and ΔΔC-peptide (Figure 2B). Based on previous literature (28, 29), we decided that the cutoff of a ΔΔC-peptide ≥ 0.1 ng/mL is likely to be of clinical significance for subjects with Wolfram syndrome. This fit the overall distribution of data, as 5 subjects (subject ID 4, 8, 10, 17, and 22) met this criterion. They represented 26.3% of the total study population. These subjects included 3 adult and 2 pediatric individuals. The ΔΔC-peptide ≥ 0.1 ng/mL cutoff was henceforth used to classify subjects as responders versus nonresponders.

There were no significant differences between responders and nonresponders in terms of HbA1c at the beginning (7.1% ± 0.2% and 7.5% ± 0.2%, respectively, *P* value 0.49) of the study and after 6 months of treatment (7.1% ± 0.3% and 7.6% ± 0.3%, respectively, *P* value 0.42) (Table 5). Prior to treatment with dantrolene, responder subjects had higher fasting C-peptide compared with nonresponders (0.47 ± 0.17 ng/dL compared with 0.18 ± 0.03 ng/dL, *P* value 0.03). By design, responder subjects demonstrated statistically significant increases in fasting C-peptide (0.53 ± 0.17 ng/dL to 0.15 ± 0.006 ng/dL), stimulated C-peptide (1.20 ± 0.33 ng/dL to 0.38 ± 0.05 ng/dL, *P* value 0.003), and ΔC-peptide (0.67 ± 0.17 ng/dL to 0.23 ± 0.02 ng/dL, *P* value 0.002) after the 6-month treatment period (Table 5). There were no significant differences between responders and nonresponders in terms of C-peptide/glucose ratios (Supplemental Figure 3 and Supplemental Table 3).

#### Markers of visual acuity.

To evaluate the effect of dantrolene treatment on visual acuity and vision-related quality of life, participants underwent ophthalmologic examination at screening, at baseline, and after 6 months of dantrolene treatment. No significant changes in visual acuity were observed across subjects or age groups as a function of dantrolene treatment (Figure 3B and Tables 3–5). Of note, subject 12 had a LogMAR = 3 throughout the study period, which equates to functional blindness. As a result, subject 12’s data were excluded from analysis. Supplemental Figure 4 demonstrates the LogMAR data including subject 12. Subgroup analyses did not identify any significant differences between adult and pediatric subjects or subjects deemed nonresponders or responders. Correspondingly, subjects reported no significant improvements in vision-related quality of life as measured by the National Eye Institute’s 25-item Visual Function Questionnaire (NEI VFQ-25) (Supplemental Table 4).

Linear regression analysis was performed comparing ΔΔC-peptide with LogMAR. This resulted in a slight, but statistically significant, negative correlation (*R*^2^ 0.136, *P* value 0.023) (Supplemental Figure 5A).

#### Disease severity and quality of life.

Overall disease severity was assessed in subjects prior to and 6 months after starting dantrolene treatment via WURS assessment (30). There were no differences in total WURS disease severity, or mean physician-rated physical exam scores, across the 6 months of the study (Figure 3, C and D, and Tables 3–5). Similarly, pediatric subjects displayed no significant changes in physical or psychosocial health domains as measured by the Pediatric Quality of Life Inventory (PedsQL) between screening and 6 months of dantrolene therapy (Supplemental Table 5), and adult subjects did not show differences in physical or mental health metrics when assessed by the SF-36v2 (Supplemental Table 6).

Similar to the LogMAR data, linear regression analysis was performed correlating ΔΔC-peptide to total WURS and physician-rated WURS. These regression plots appeared to demonstrate a negative correlation but did not reach statistical significance (*R*^2^ 0.085, *P* value 0.117 and *R*^2^ 0.021, *P* value 0.444, for total WURS and physician-rated WURS, respectively) (Supplemental Figure 5, B and C).

#### Exploratory biomarker levels.

It has been shown that ER stress induces sterile inflammation and oxidative stress (31–33). Therefore, we assessed expression levels of inflammatory cytokines and isoprostane, an established marker of oxidative stress in study subjects prior to and 6 months after starting dantrolene treatment via WURS assessment (34–36) (Supplemental Table 7). As compared with levels previously reported in heathy subjects (35–37), levels of IFN-γ, TNF-α, IL-1β, MIP-1β, MIP-3α, IL-21, and isoprostane were higher in study subjects with Wolfram syndrome. Among those, a statistically significant decrease in IL-1β and IL-21 levels was seen after 6 months of treatment with dantrolene sodium.

## Discussion

In this study, we evaluate the safety and tolerability of dantrolene sodium as a therapeutic approach for Wolfram syndrome. Our previous preclinical studies showed that dantrolene improves β cell and neuronal cell survival in mouse and patient iPSC models of this disease (8). To translate these findings to humans, we conducted the first clinical trial in pediatric and adult subjects with Wolfram syndrome in a 6-month study of dantrolene sodium (NCT02829268). We identified a tolerable range of oral dantrolene dosing of 0.5 mg/kg/d to 2 mg/kg/d for pediatric subjects and 50 mg/d to 100 mg/d for adults. We did not find any significant changes in their baseline medications or drug interaction that might affect the efficacy. Overall, dantrolene was very well tolerated, and aside from mild fatigue and diarrhea, no clinically significant adverse events were reported.

Admittedly, this proof-of-concept study ran into many of the same issues that plague early clinical trials for rare diseases (21). As the incidence of Wolfram syndrome is so rare, it is difficult to recruit a large enough sample size in order to detect a statistically significant difference in secondary measures of β cell function, visual acuity, or quality of life. To aid with recruitment, our study team collaborated with existing natural history studies and patient organizations. Another challenge facing this study is the vast clinical heterogeneity seen in the spectrum of individuals with Wolfram syndrome (38). Independent of age, some individuals are more severely affected or progress more rapidly than others. Our hypothesis is that these differences may be based on the severity of the *WFS1* gene variants. For example, individuals with missense pathogenic variants may have a less severe course compared with individuals with large deletions or nonsense pathogenic variants. This clinically and genetically heterogeneous population makes it more challenging to infer cause-and-effect relationships when studying a potential drug. As a result, our strategy has been to target the underlying cellular defect (ER calcium depletion) that is unified among all patients with Wolfram syndrome (39).

With the above challenges in mind, there remain additional shortcomings of this study. As it is an uncontrolled study, certain parameters measured are susceptible to confounding by the placebo effect. Due to the small sample sizes, there were no statistically significant differences in β cell function or disease severity. For these reasons, this study does not posit that dantrolene improves β cell function or disease severity. Instead, it identifies safe doses for treatment of adult and pediatric subjects with Wolfram syndrome, highlights the side effect profile of dantrolene in this population, and argues that further investigation of dantrolene, or investigational agents with a similar mechanism of action, are warranted in a randomized, double-blind, placebo-controlled study. With this caveat in mind, this study also suggests that dantrolene requires further investigation in the context of β cell function and neurodegeneration.

Perhaps the most salient question arising from this study is whether dantrolene improves human β cell function in Wolfram syndrome. While a controlled human study is required to assess dantrolene’s efficacy at improving β cell function, pediatric subjects in our study started to exhibit a trend toward higher stimulated C-peptide levels after 6 months of sustained dantrolene treatment. Adult subjects, in contrast, showed a negligible increase in mean stimulated C-peptide levels throughout their duration of dantrolene treatment. These data suggest that dantrolene may be more effective in pediatric subjects, possibly because these subjects have a larger surviving subpopulation of functional β cells during this initial phase of their disease process. Evidently, adult subjects also secrete very low levels of insulin, but dantrolene did not seem to significantly enhance β cell function in this group.

The significance of these small elevations in C-peptide is quite interesting when comparing Wolfram syndrome with type 1 diabetes. Recently, there has been a growing body of literature suggesting that there is clinical benefit from a very small degree of residual β cell function. Oram and colleagues published a population-based study in the United Kingdom, demonstrating that 8% of subjects had a urinary C-peptide/creatinine ratio ≥ 0.2 nmol/mmol (29). This study and another follow-up study in 2019 demonstrated that this persistent microsecretion of C-peptide is associated with fewer complications of diabetes and less hypoglycemia (28). Contrasting these populations, subjects with Wolfram syndrome tend to have much higher C-peptide compared with type 1 diabetes. Notably the preserved C-peptide group in the 2019 study had a mean stimulated C-peptide of 114 pmol/L (0.3443 ng/mL). This is compared with a mean stimulated C-peptide of 0.52 ng/mL (205 pmol/L) in our study population with Wolfram syndrome. These data suggest that small, but statistically insignificant, increases in C-peptide may be clinically significant. We suggest that future studies of dantrolene or similar agents track changes in total daily insulin dose (with percentage basal versus bolus) and analyze continuous glucose monitor tracings (i.e., time in range, time in hypoglycemia, and standard deviation).

As previously reported, markers of inflammatory cytokines and oxidative stress, such as IFN-γ, IL-1β, TNF-α, and isoprostane, were elevated in subjects with Wolfram syndrome (31–33, 35). Biomarkers for neurodegeneration, such as neurofilament light chain and myelin basic protein, have been assessed in patients with Wolfram syndrome before (31, 40). Further studies on such biomarkers and their relationships in phenotypes of patients will lead to an identification of surrogate biomarkers for clinical outcomes in patients with Wolfram syndrome.

We postulate that efficacy of dantrolene may be linked to the nature of the *WFS1* mutations in the individual subjects. Over time, and with further experience with dantrolene, perhaps dantrolene can be part of a personalized medicine approach for patients with Wolfram syndrome.

In summary, this study suggests that dantrolene sodium is safely tolerated by subjects with Wolfram syndrome. Although the study was small, a select few subjects seemed to have improvements in β cell function. Therefore, this study justifies further investigation into using dantrolene sodium and other new medications targeting ER stress for the treatment of Wolfram syndrome.

## Methods

### Study protocol.

Dantrolene sodium was dispensed to the study subjects via Washington University’s clinical trials pharmacy. Subjects were instructed to take the dantrolene by mouth. Subjects enrolled in this study underwent a run-in period for dose maximization (Figure 1A). Adult subjects were started on up to 25 mg dantrolene daily for 7 days, then doubled in dose on a weekly basis up to a maximum of 200 mg dantrolene daily. Pediatric subjects (<18 years old) were started on up to 0.5 mg/kg dantrolene daily (maximum 25 mg) for 7 days, then doubled in dose on a weekly basis up to a maximum of 2 mg/kg dantrolene daily (maximum 200 mg), with no dose change if weight fell within ±3% of the original dosing weight. Dosing calendars were maintained by the study subjects to ensure adherence to the study drug.

### Safety assessment and outcomes measures.

Baseline screening procedures included complete physical exam, standard clinical laboratory tests (serum chemistry, liver function tests, hematology, and urinalysis), and 12-lead ECG. Subjects underwent formal visual acuity testing by the coauthors of the study who are either optometrists or ophthalmologists. At baseline, each subject underwent the WURS (30). The 30-minute MMTT was performed to assess baseline β cell functions as described before (24). The mixed meal consisted of 6 mL/kg (maximum 360 mL) of Boost (Nestle) consumed over a maximum of 5 minutes. After the overnight fasting, blood for glucose and C-peptide measurement was drawn at time 0 (fasting) and 30 minutes after the Boost. If a subject’s fasting glucose exceeded 250 mg/dL, the test was not performed, but fasting glucose and C-peptide were obtained. An ECG was performed before and 4 hours after the first dose of dantrolene was administered during the run-in period, then again at 2 months and 6 months. Best-corrected visual acuity was assessed by Snellen optotype and converted to LogMAR score (41). Vision-related quality of life was assessed in all subjects at screening and after 6 months of dantrolene by the NEI VFQ-25 (42). Functional activities of daily living were assessed in pediatric subjects by the PedsQL (https://www.pedsql.org/) (43), while the SF-36v2 (https://www.optum.com/) was used to measure self-reported functional health and well-being of adults at baseline and after 6 months of dantrolene therapy (44). If no safety concerns were identified at screening, subjects began the 3-week dose maximization period of dantrolene sodium. All baseline screening procedures were repeated again at 6 months of treatment to ensure subject safety and assess dantrolene tolerability. Grip strength was measured at each visit bilaterally using a digital hand dynamometer (CAMRY). A final safety follow-up visit was conducted at 28 days (±7 days) after the last outcome measure evaluation in order to collect additional information on adverse events, concomitant medications, therapies, and procedures. For subjects who discontinued the study prior to the first outcome measure evaluation, safety follow-up visit was conducted within 28 days (±7 days) after the last administration of dantrolene sodium.

### Labs and biomarker levels.

Comprehensive metabolic panel, HbA1c, C-peptide, and complete blood count (CBC) were performed by the Core Laboratory for Clinical Studies at Washington University in St. Louis. This is a Clinical Laboratory Improvement Amendments–certified and College of American Pathologists–accredited laboratory. The CMP and HbA1c were performed on a Roche c501 automated analyzer with commercially available kits from Roche. Specifically, HbA1c used the Tina Quant, Gen 3 assay. The C-peptide was performed on Roche’s e601 automated analyzer, which uses a sandwich electrochemiluminescence immunoassay. The precision, as measured by coefficient of variation (CV; %) of these assays, is between 1.2% and 3.8%. CBC was performed on the Beckman Coulter UniCell DxH600, an automated hematology analyzer. Ammonia, urine human chorionic gonadotropin, serum osmolality, and urine analysis were sent to Quest Laboratories for testing.

Inflammatory cytokine levels were assessed at the immune-monitoring laboratory at Washington University. The MILLIPLEX MAP Human High Sensitivity T Cell Magnetic Bead Panel (HSTCMAG28SMPX21; MilliporeSigma) simultaneously measures 21 analytes (fractalkine, GM-CSF, IFN-γ, IL-1β, IL-2, IL-4, IL-5, IL-6, IL-7, IL-8, IL-10, IL-12p70, IL-13, IL-17A, IL-21, IL-23, ITAC, MIP-1α, MIP-1β, MIP-3α, and TNF-α) using a magnetic bead–based fluorescence sandwich ELISA. Samples were thawed on ice, centrifuged at 15,000 RCF for 5 minutes to remove particulates, and then added in duplicate wells to prepared beads, according to the manufacturer’s protocol. A Luminex FLEXMAP3D instrument was used to collect and measure the MFI of 50 beads per analyte per well. The assay contained recombinant protein standards for each analyte and a quality control (QC) sample. The interassay precision was assessed by percentage recovery of each standard analyte, percentage CV of replicate wells, and percentage recovery of the QC sample. Percentage recoveries were within 75%–120% of the expected concentration for all analytes and the QC. The percentage CV of replicates was less than 25% and generally less than 10% for most samples. A 5-parameter logistic function with 1/*y*^2^ weighting was fit using Belysa v.1 analysis software (MilliporeSigma). The lower limit of quantitation was defined by the software for each analyte.

F2-isoprostanes were analyzed in the Eicosanoid Core Laboratory at Vanderbilt University Medical Center. For analysis, 1 mL plasma was diluted to a volume of 10 mL by the addition of 0.01N HCl. The internal standard (1.0 ng, [^2^H_4_]-15-F_2t_-IsoP) was added, and the solution was adjusted to pH 3 with 1N HCl. The sample was then applied to a C-18 Sep-Pak cartridge (Waters) that had been prewashed with 5 mL methanol and 5 mL 0.01N HCl. The cartridge was washed with 10 mL 0.01N HCl, followed by 10 mL heptane, and compounds were then eluted with 10 mL ethyl acetate/heptane (50:50, v/v). The eluate was dried under nitrogen. Analytes were converted to the pentafluorobenzyl (PFB) esters by the addition of 40 μL of a 10% solution of pentafluorobenzyl bromide in acetonitrile and 20 μL of a solution of 10% *N,N*-Diisopropylethylamine in acetonitrile and allowed to incubate for 30 minutes at 37°C. Reagents were dried under nitrogen and the residue was reconstituted in 30 μL chloroform and 20 μL methanol and chromatographed on a silica TLC plate to 13 cm in a solvent system of chloroform/methanol (93:7, v/v). The methyl ester of PGF_2α_ was chromatographed on a separate lane and visualized with 10% phosphomolybdic acid in ethanol by heating. The R_f_ of PGF_2α_ methyl ester in this solvent system is 0.15. Compounds migrating in the region 1 cm below the PGF_2α_ standard to 1.0 cm above the standard were scraped from the TLC plate, extracted with 1 mL ethyl acetate/ethanol (85:15, v/v), and dried under nitrogen. Following TLC purification, compounds were converted to trimethylsilyl (TMS) ether derivatives by addition of 20 μL *N,O*-bis(trimethylsilyl)trifluoroacetamide and 10 μL dimethylformamide. The sample was incubated at 37°C for 10 minutes and then dried under nitrogen. The residue was dissolved for gas chromatography/mass spectrometry (GC/MS) analysis in 20 μL undecane that had been stored over a bed of calcium hydride. GC/negative ion chemical ionization–MS (GC/NICI-MS) was carried out on an Agilent 5973 Inert Mass Selective Detector that was coupled with an Agilent 6890n Network GC system that is interfaced with an Agilent computer. GC was performed using a 15 m, 0.25 μm film thickness, with DB-1701 fused silica capillary column (J and W Scientific). The column temperature was programmed from 190°C to 300°C at 20°C per minute. The major ion generated in the NICI mass spectrum of the PFB ester, TMS ether derivative of F_2_-IsoPs, was the *m/z* 569 carboxylate anion [M-181 (M-CH_2_C_6_F_5_)]. The corresponding ion generated by the [^2^H_4_]-15-F_2t_-IsoP internal standard was *m/z* 573. Levels of endogenous F_2_-IsoPs in a biological sample are calculated from the ratio of intensities of the ions *m/z* 569 to *m/z* 573. Employing this assay, the lower limit of detection of F_2_-IsoPs is 4 pg using an internal standard with a blank of 3 parts per thousand. The precision of this assay in biological fluids is +6% and the accuracy 94%. The normal range of F_2_-IsoPs in plasma is 35 ± 6 pg/μL (34–36).

### Statistics.

Secondary outcome measures (C-peptide, LogMAR, WURS, HbA1c, BMI, glucose, proinsulin, insulinogenic index, AUC C-peptide/AUC glucose, proinsulin/C-peptide) were reported as the median and interquartile range. When analyzing differences among the same group (all subjects, adult, pediatric, nonresponders, and responders) at 0 and 6 months, we used the Wilcoxon signed-rank test, using SciPy (https://www.scipy.org) and pandas (https://pandas.pydata.org/index.html) programming libraries. Subjects who dropped out of the study or who did not complete a secondary outcome measure were excluded from these analyses. Subject 12 had a LogMAR of 3 (functionally blind); therefore, this subject’s LogMAR score was excluded from the analysis. The Mann-Whitney *U* test was used when comparing between groups (adult vs. pediatric subjects or nonresponders vs. responders), also using SciPy and pandas programming libraries. Linear regression analyses (including *R*^2^ and *P* values) were constructed using the ordinary least squares method using the statsmodels (https://www.statsmodels.org/devel/about.html#about-statsmodels) programming library. A *P* < 0.05 was considered significant for all analyses. Figures were constructed with matplotlib (https://matplotlib.org) and seaborn (https://seaborn.pydata.org) programming libraries or GraphPad Prism 8 software (https://www.graphpad.com/scientific-software/prism/). Receiver operator characteristic curves and calculations were constructed using GraphPad Prism 8 software.

### Study approval.

Subjects, and their parent or legal guardian, as appropriate, provided written, informed consent before participating in this study, which was approved by the Human Research Protection Office at Washington University School of Medicine in St. Louis (IRB ID #201607006).

Subjects who met all of the following criteria were eligible for enrollment: first, a definitive diagnosis of Wolfram syndrome, as determined by the following: a) documented functionally relevant recessive mutations on both alleles of the *WFS1* gene or b) a dominant mutation on 1 allele of the *WFS1* gene based on historical test results (if available) or from a qualified laboratory at screening; second, the subject was at least 5 years of age (biological age) at the time of written informed consent; and third, the subject, subject’s parent(s), or legally authorized guardian(s) must have voluntarily signed an IRB/Independent Ethics Committee-approved informed consent form after all relevant aspects of the study have been explained and discussed with the subject. The guardians’ consent and subjects’ assent, as relevant, were obtained.

## Author contributions

FU designed the study. BAM, NHW, TSP, RCB, and TH advised on the design of the study. ANS recruited participants and ANS and SH managed the study. TSP, RCB, JH, LT, SIS, BAM, NHW, GPVS, and FU examined subjects. SH, CMB, and KK collected the data. CMB, DA, SIS, TH, CO, and FU analyzed the data. TH advised on the statistical analysis. DA and SIS wrote the first draft of the manuscript and all the authors revised it critically and approved the final version. DA and SIS are co–first authors: DA started the analysis of the data and SIS completed it.

## Supplementary Material

Supplemental data

## Figures and Tables

**Table 1:**
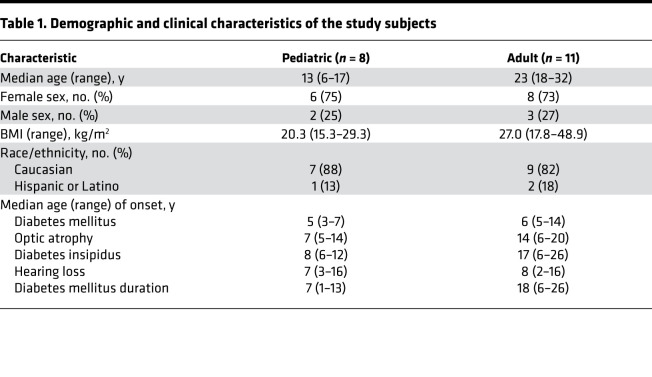
Demographic and clinical characteristics of the study subjects

**Table 2:**
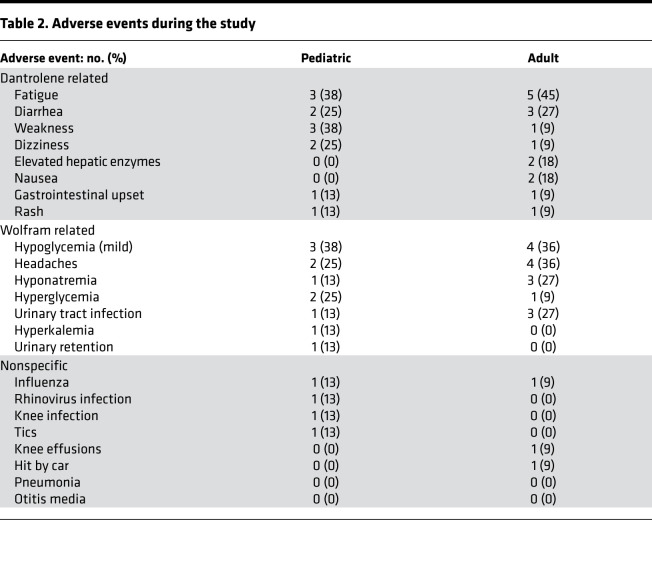
Adverse events during the study

**Table 3:**
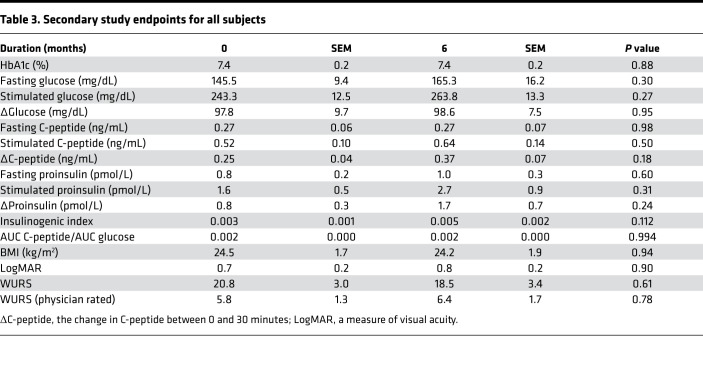
Secondary study endpoints for all subjects

**Table 4:**
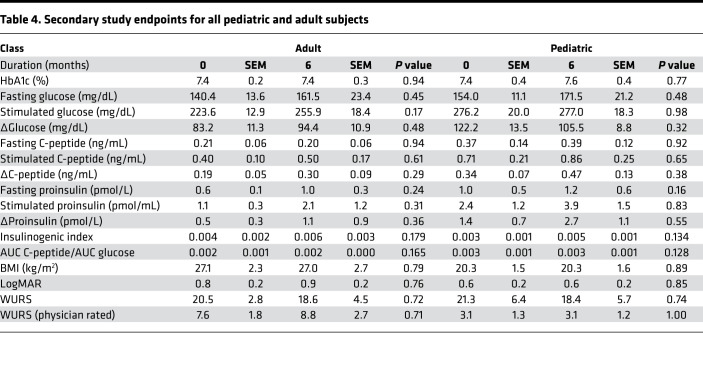
Secondary study endpoints for all pediatric and adult subjects

**Table 5:**
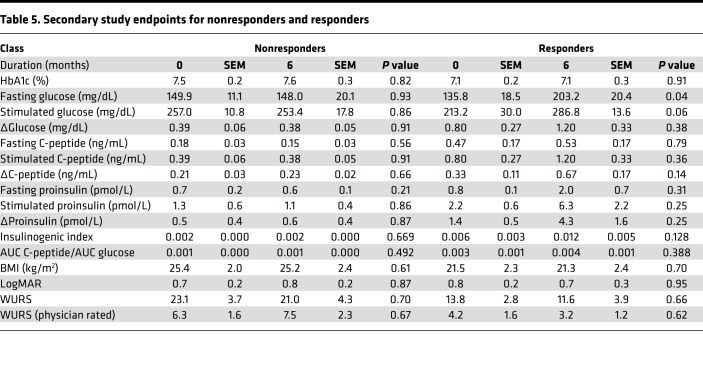
Secondary study endpoints for nonresponders and responders

## Version 1 06/29/2021

In-Press Preview

## Version 2 08/09/2021

Electronic publication
