# Supplementary material for: A phase Ib/IIa clinical trial of dantrolene sodium in patients with Wolfram syndrome
Source: JCI Insight. 2021 Aug 9;6(15):e145188. doi: 10.1172/jci.insight.145188 (PMC8410026; doi:10.1172/jci.insight.145188)
Supplement: Supplemental data [file jciinsight-6-145188-s152.pdf]

Supplementary Information

Supplementary Figure 1. Grip strength of each study subject during the study. A) Right hand B) Left hand.

Supplementary Figure 2. C-peptide data from each study subject over the course of the study. Blue represents baseline (fasting) C-peptide. Orange represents 30-minute (stimulated) C-peptide. C-peptide is in ng/mL.

Supplementary Figure 3. Additional markers of β-cell function. A) Proinsulin collected during a mixed meal tolerance text. B) Insulinogenic Index. C) Area Under the Curve (AUC) C-peptide /AUC Glucose. D) C-peptide to Glucose Ratio All study subjects are broken down into adult and pediatric subgroups. Light boxes represent fasting results, while dark boxes represent 30-minute (stimulated) values. Responders are differentiated from non-responders by having a change in Δ C-peptide (ΔΔ C-peptide) ≥ 0.1 ng/mL over the course of the study.

Supplementary Figure 4. LogMAR visual acuity plot including subject 12. Subject 12 was excluded from the analysis as they are blind with a LogMar = 3.

Supplementary Figure 5. Linear regression analysis. Linear regression analysis comparing ΔΔ C-peptide to (A) LogMAR visual acuity, (B) total WURS, and (C) physician rated WURS. R2 and p-values are demonstrated in the top right corner of each panel.

Supplementary Figure 1

A

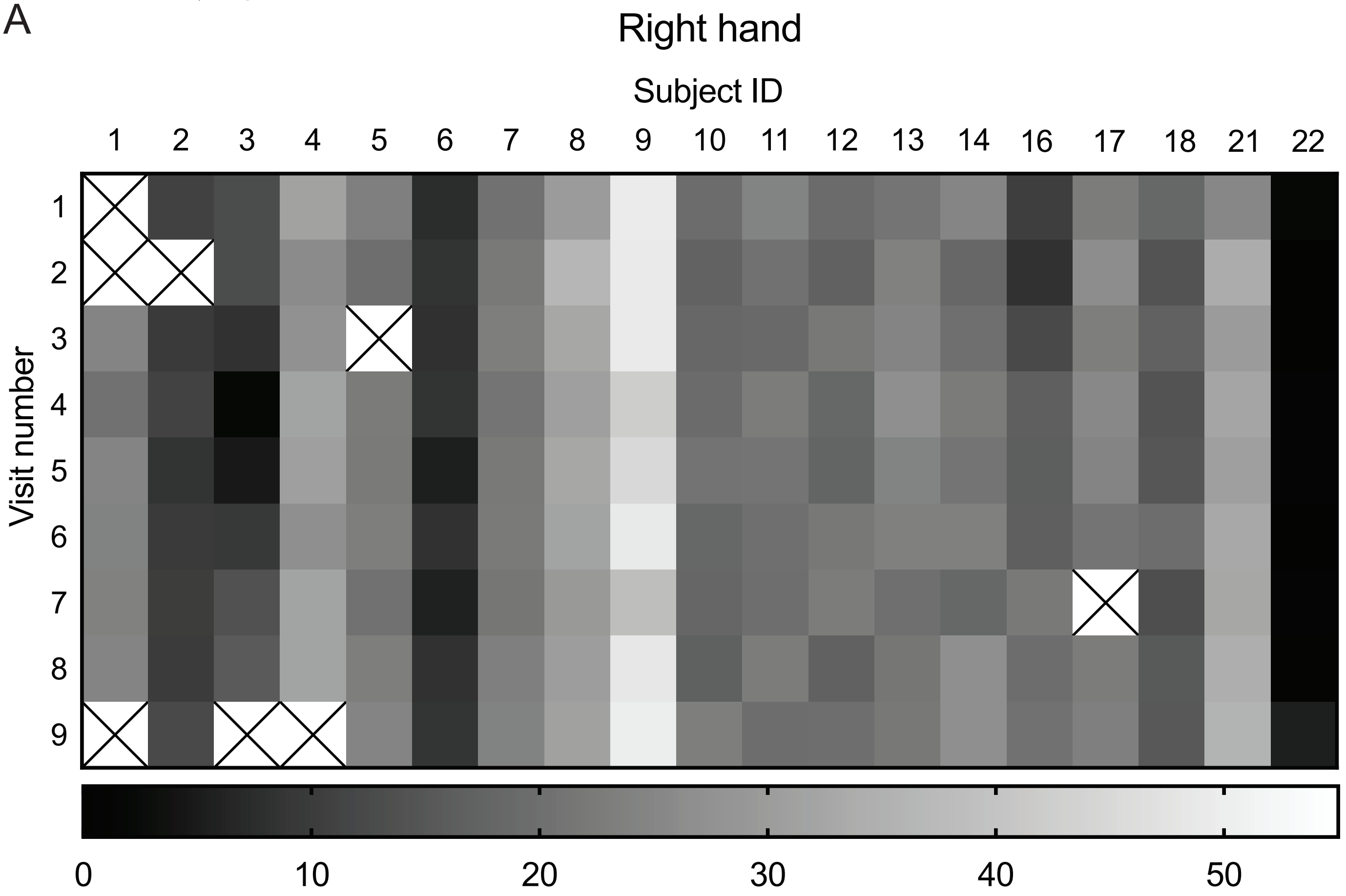

B

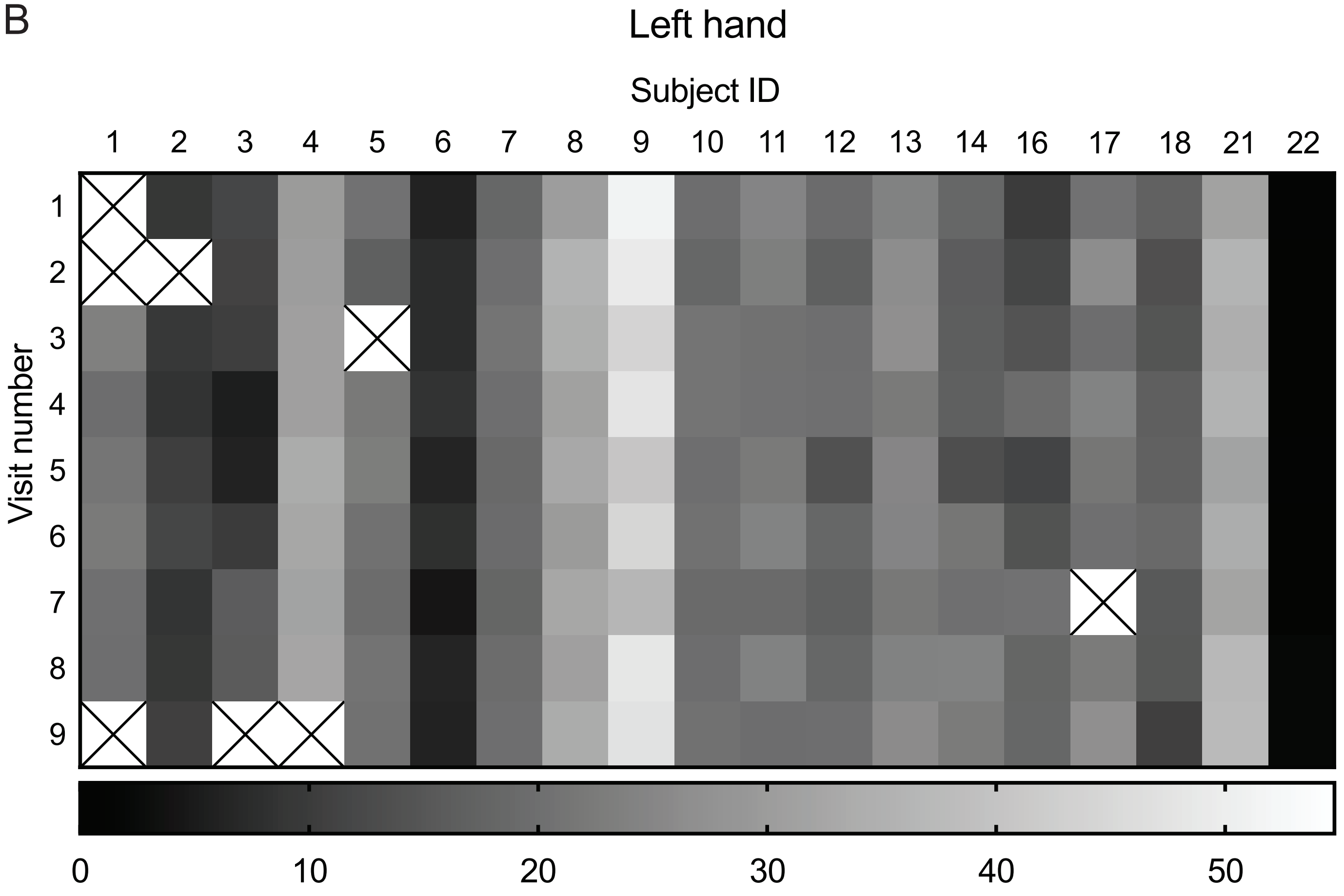

Supplementary Figure 2

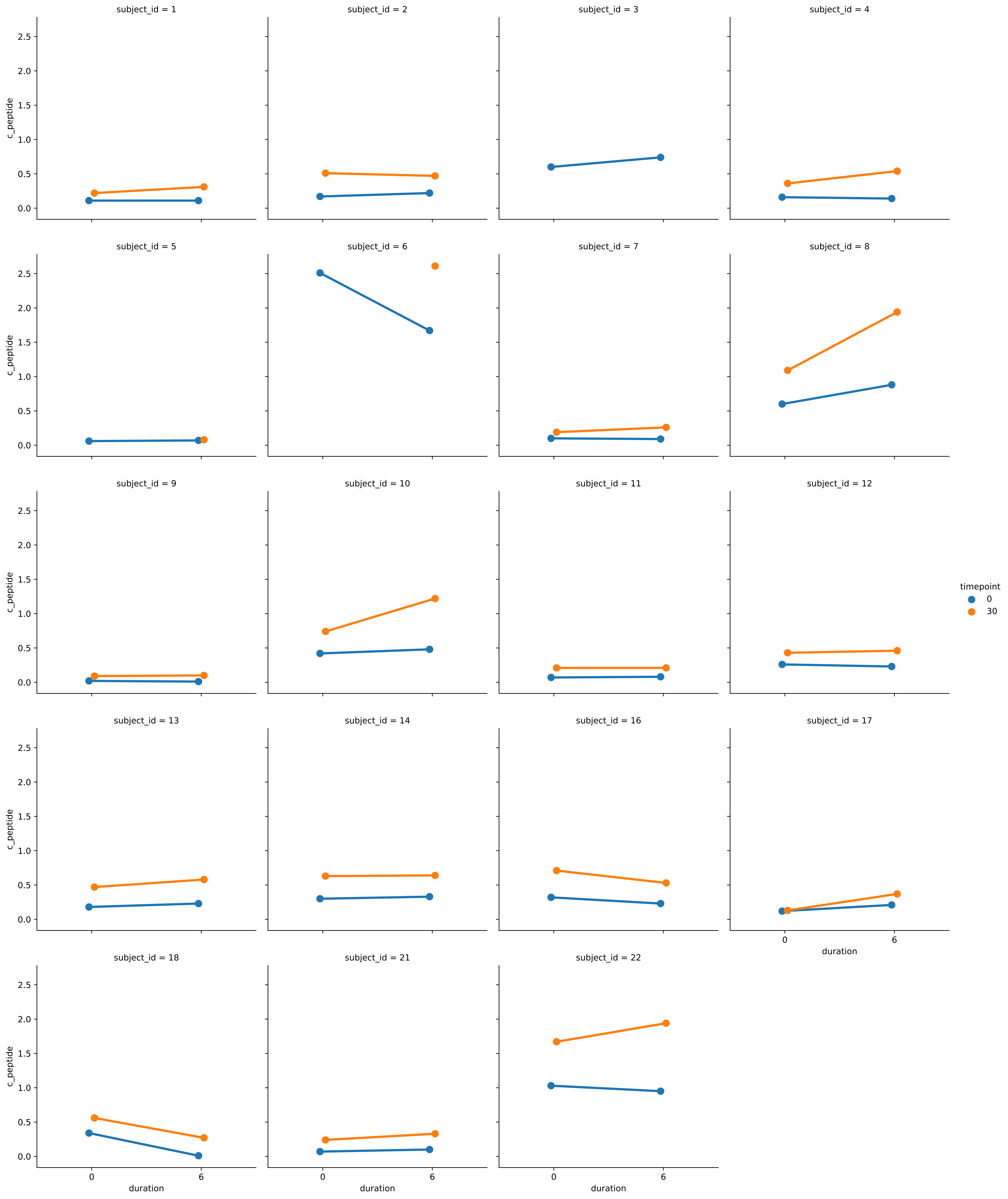

Supplementary Figure 3

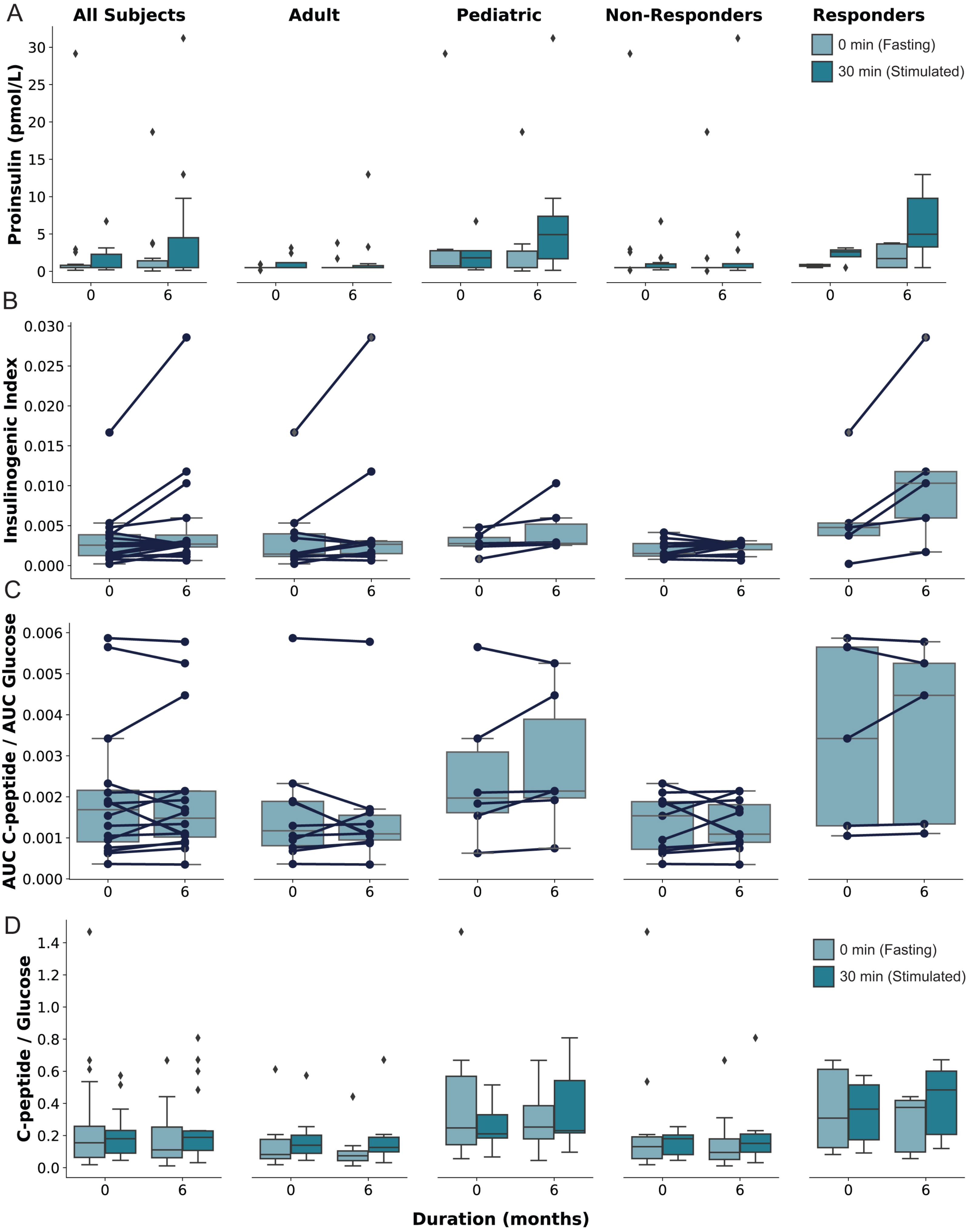

Supplementary Figure 4

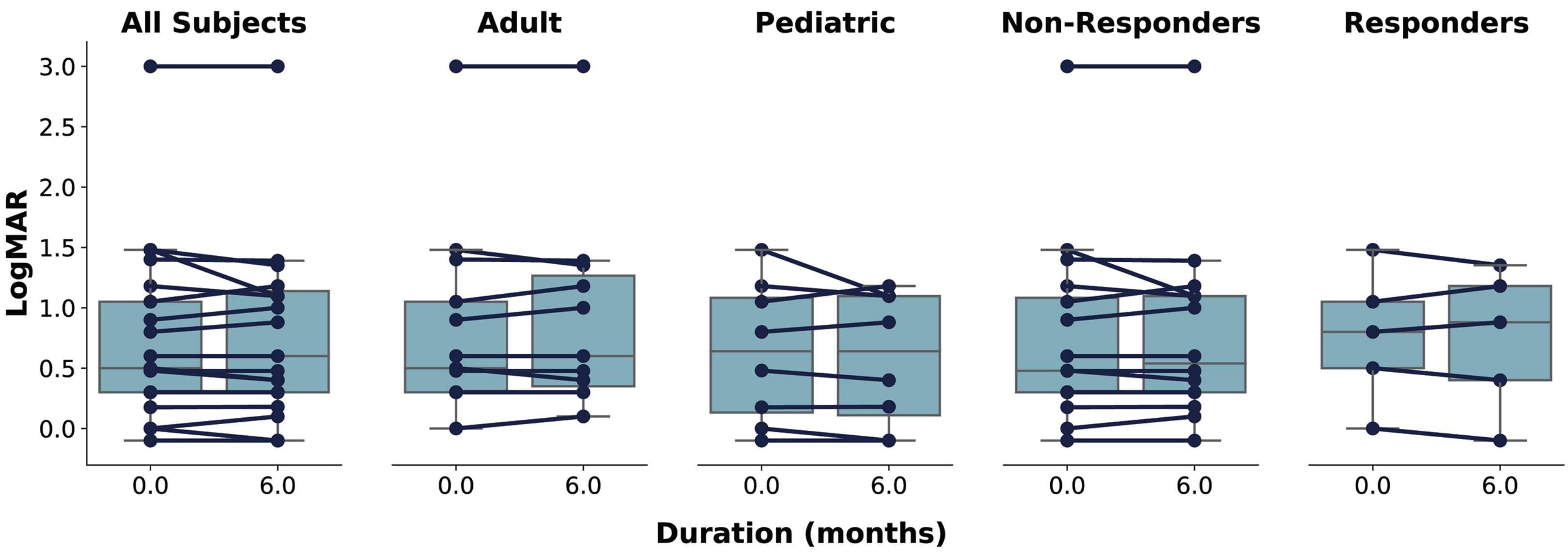

Supplementary Figure 5

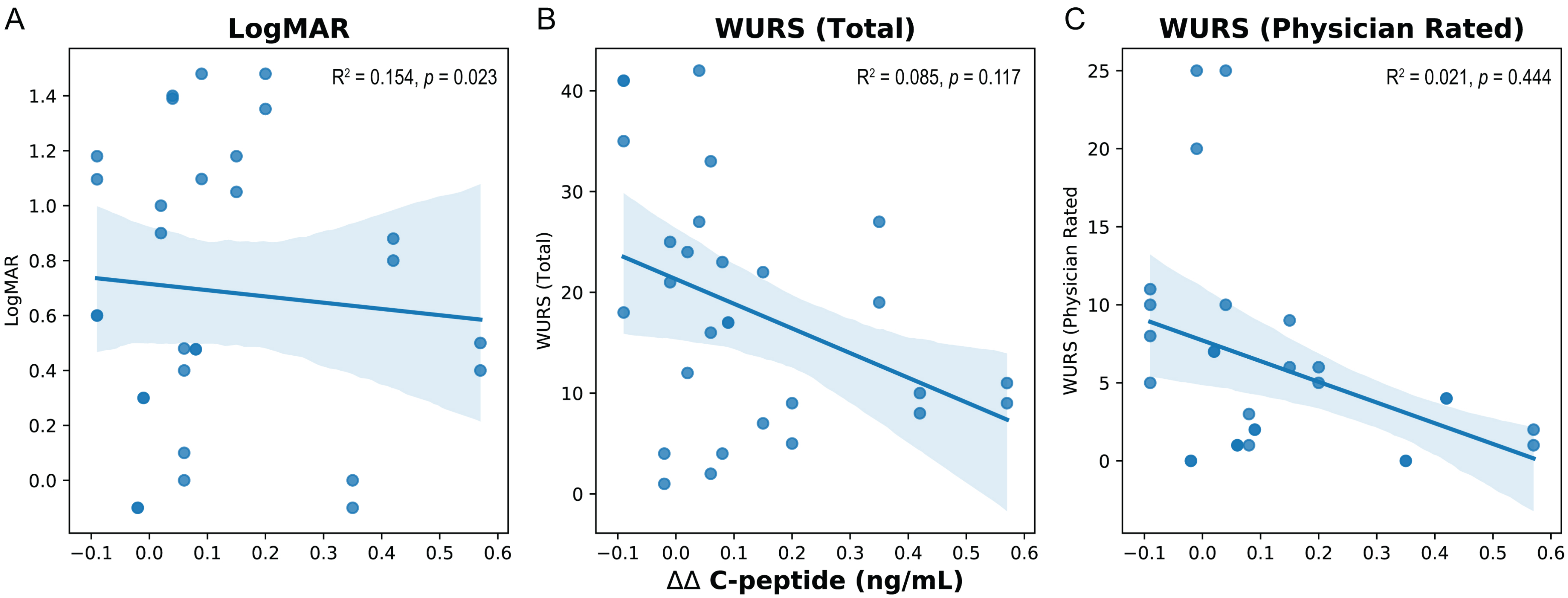

	WFS1		Age at Diagnosis				Duration (yrs)
Subject ID	Allele 1	Allele 2	Diabetes Mellitus	Optic Atrophy	Diabetes Insipidus	Hearing Loss	Diabetes Mellitus
1	p.(R147Gfs*17)	p.(W540*)	3	7	12	9	13
2	p.(L200Rfs*87)	p.(E752*)	5	5	7	7	7
3	p.(E169K)	p.(E169K)	5	6	6	6	4
4	p.(V412Sfs*29)	p.(V415del)	5	6	6	6	20
5	p.(L200P)	p.(R232P)	5	15	--	16	18
6	p.(L200P)	p.(R232P)	7	--	--	3	1
7	p.(K253*)	p.(F883Sfs*68)	7	20	22	--	19
8	p.(E202G)	p.(D211N)	14	13	--	--	6
9	p.(G107E)	p.(R629W)	6	17	17	7	23
10	p.(F883Sfs*68)	--	6	9	10	16	11
11	p.(H313Y)	--	4	13	--	2	16
12	p.(E273*)	p.(W613*)	6	8	11	9	12
13	p.(E202G)	p.(F350del)	7	14	--	--	8
14	p.(E202G)	p.(F350del)	5	--	--	--	7
16	p.(L282P)	p.(W613*)	6	18	17	16	17
17	p.(S790*)	p.(L567_F568del)	7	12	--	--	26
18	p.(E169K)	p.(V415del)	9	15	?	8	18
21	p.(F413_V415del)	p.(F883Sfs*68)	5	14	16	--	17
22	p.(R42Efs*101)	p.(F883Sfs*68)	4	--	--	--	2

Supplementary Table S1. Genetic and Clinical Characteristics of the Study Subjects

Indication	Medication Names		No. (%) of Patients	
Indication	Pediatric	Adult	Pediatric (n=8)	Adult (n=11)
Diabetes	Humalog, Insulin, Lantus, Levemir, Novolog	Calcium, Desmopressin, Humalog, Insulin, Iron, Lantus, Metaformin, Novolog, Victoza	8 (100.0)	11 (100.0)
Supplement	CoQ10, Fiber-Con, Fiber Gummies, Fish Oil, Idebenone, Iron, Jucie Plus, Multivitamin, NAC, Nicotenamide, Omega 369, TUDCA, Vitamin B12	Calcitriol, Cholecalciferol, CoQ10, Cranberry Extract, DHA 500, Iron, Minrex, Multivitamin, Navitol, Sodium Chloride, Vitamin B-Complex, Vitamin C, Vitamin D, Vitamin D3, Vitamin E	6 (75.0)	9 (81.8)
Behavior/Mood	Ambilify, Citalopram, Rispedirone, Vistal, Wellbutrin, Zoloft	Ativan, Bupropion ER, Focalin, Guanfacine, Hydroxyzine Pamoate, Lamotrigine, Paxil, Setraline, Vyvanse	3 (37.5)	6 (54.5)
Pain	Acetominophen, Ketorolac, Magnesium Citrate, Prochlorperazine, Topiramate	Acetominophen, Ibuprofen, Keppra, Lamotrigene, Mefenamic Acid	1 (12.5)	5 (45.5)
GI Problems	Dicyclomin, Pepcid Complete, Pentasa, Zantac	Metocloperamide, Nexium, Nizatidine, Omeprazole, Probiotic	2 (25.0)	4 (36.4)
Antibiotic	Amoxicillin, Azithromycin, Azo, Cefepime	Ciprofloxacin, Doxyclycline Hyclate, Macrobid	3 (37.5)	2 (18.2)
Constipation	Miralax, Senna	Docusate, Linzess, Polyethylene Glycol	2 (25.0)	3 (27.3)
Birth Control	-	Camrese, Glidess, JunetFe, Levonorgestrel, Ortho Tri-Cyclen, Tri-Estarylla, Trisprintec	-	5 (45.5)
Nocturnal Enuresis	Desmopressin	Desmopressin	3 (37.5)	1 (9.1)
Antiviral	Tamiflu	Tamiflu, Oseltamivir, Valaciclovir	1 (12.5)	2 (18.2)
Allergies	Allegra, Claritin	Zyrtec	2 (25.0)	1 (9.1)
Bladder Problems	Azo	Vesicare	1 (12.5)	1 (9.1)
Nausea/Vomiting	Prochlorperazine	Ondansetron	1 (12.5)	1 (9.1)
Diarrhea	Immodium	Linzess	1 (12.5)	1 (9.1)
Low Testosterone	-	Androgel	-	2 (18.2)
Sleep Aid	-	Melatonin, Modafinil	-	2 (18.2)
Tinea	-	Ketoconazole	-	1 (9.1)
Hypothyroid	-	Levothyroxine	-	1 (9.1)
Hypertension	-	Guanfacine, Lisinopril, Losarten	-	1 (9.1)
High Cholesterol	-	Rosuvastatin	-	1 (9.1)
Appetite Stimulant	-	Cyproheptadine	-	1 (9.1)
Nasal Drainage	-	Ipravent	-	1 (9.1)
Cough Suppressant	-	Promethazine DM Syrup	-	1 (9.1)
Hyponatremia	Sodium Chloride	-	1 (12.5)	-
Hypoglycemia	Glucagon	-	1 (12.5)	-
Molluscum	Cetirizine, Zyrtec	-	1 (12.5)	-
Rash	Benadryl, Cetirizine, Diphenhydramine, Triamcinolone, Zyrtec	-	1 (12.5)	-
Anemia	Iron	-	1 (12.5)	-
Trigeminal Neuralgia	Carbamazepine, Naproxen	-	1 (12.5)	-

Table S2. Concomitant Medications Used Throughout Trial

Duration (Months)	0					6					0					6				
**Class**	**Adults**	**SEM**	**Pediatrics**	**SEM**	***p*-value**	**Adults**	**SEM**	**Pediatrics**	**SEM**	***p*-value**	**Non-Responders**	**SEM**	**Responders**	**SEM**	***p*-value**	**Non-Responders**	**SEM**	**Responders**	**SEM**	***p*-value**
BMI (kg/m^2)	27.1	2.3	20.3	1.5	0.05	27.0	2.7	20.3	1.6	0.07	25.4	2.0	21.5	2.3	0.32	25.2	2.4	21.3	2.4	0.37
HbA1c (%)	7.4	0.2	7.4	0.4	0.97	7.4	0.3	7.6	0.4	0.70	7.5	0.2	7.1	0.2	0.49	7.6	0.3	7.1	0.3	0.42
Fasting Glucose (mg/dL)	140.4	13.6	154.0	11.1	0.50	161.5	23.4	171.5	21.2	0.78	149.9	11.1	135.8	18.5	0.51	148.0	20.1	203.2	20.4	0.12
Stimulated Glucose (mg/dL)	223.6	12.9	276.2	20.0	0.04	255.9	18.4	277.0	18.3	0.46	257.0	10.8	213.2	30.0	0.11	253.4	17.8	286.8	13.6	0.26
Δ Glucose (mg/dL)	83.20	11.29	122.17	13.46	0.05	94.40	10.86	105.50	8.81	0.49	107.09	7.01	77.40	26.61	0.16	105.36	6.74	83.60	18.41	0.19
Fasting C-peptide (ng/dL)	0.21	0.06	0.37	0.14	0.23	0.20	0.08	0.39	0.12	0.20	0.18	0.03	0.47	0.17	0.03	0.15	0.03	0.53	0.17	0.01
Stimulated C-peptide (ng/dL)	0.40	0.10	0.71	0.21	0.15	0.50	0.17	0.86	0.25	0.23	0.39	0.06	0.80	0.27	0.06	0.38	0.05	1.20	0.33	0.00
Δ C-peptide (ng/dL)	0.2	0.0	0.3	0.1	0.10	0.3	0.1	0.5	0.1	0.28	0.2	0.0	0.3	0.1	0.19	0.2	0.0	0.7	0.2	0.00
Fasting Proinsulin (pmol/L)	0.6	0.1	1.0	0.5	0.24	1.0	0.3	1.2	0.6	0.73	0.7	0.2	0.8	0.1	0.95	0.6	0.1	2.0	0.7	0.01
Stimulated Proinsulin (pmol/L)	1.1	0.3	2.4	1.2	0.19	2.1	1.2	3.9	1.5	0.38	1.3	0.6	2.2	0.6	0.40	1.1	0.4	6.3	2.2	0.01
Δ Proinsulin (pmol/L)	0.5	0.3	1.4	0.7	0.20	1.1	0.9	2.7	1.1	0.29	0.5	0.4	1.4	0.5	0.22	0.6	0.4	4.3	1.6	0.01
Insulinogenic Index	0.004	0.002	0.003	0.001	0.737	0.006	0.003	0.005	0.001	0.765	0.002	0.000	0.006	0.003	0.044	0.002	0.000	0.012	0.005	0.007
AUC C-peptide / AUC Glucose	0.002	0.001	0.003	0.001	0.352	0.002	0.000	0.003	0.001	0.168	0.001	0.000	0.003	0.001	0.013	0.001	0.000	0.004	0.001	0.006
Logmar Score	0.63	0.14	0.63	0.21	1.00	0.14	0.71	0.58	0.19	0.60	0.59	0.13	0.77	0.25	0.51	0.62	0.13	0.74	0.27	0.65
WURS	20.50	2.82	21.25	6.38	0.91	18.59	4.51	18.38	5.71	0.98	23.13	3.68	13.80	2.82	0.18	20.96	4.34	11.60	3.94	0.24
WURS (Physician Rated)	7.6	1.8	3.1	1.3	0.09	8.8	2.7	3.1	1.2	0.11	6.3	1.6	4.2	1.6	0.48	7.5	2.3	3.2	1.2	0.28

Supplementary Table S3. Table comparing subgroup analyses at each timepoint

	Duration of Dantrolene Treatment (months)	
	**0**	**6**
**VFQ-25 Domain**	(n=19)	(n=19)
General health	60.5 (4.4)	56.6 (4.6)
General vision	61.1 (6.4)	56.8 (5.4)
Ocular pain	81.6 (4.4)	82.2 (4.2)
Near activities	59.2 (4.7)	57.0 (4.9)
Distance Activities	65.9 (4.3)	59.6 (4.5)
Vision specific		
Social functioning	77.6 (5.0)	77.0 (5.1)
Mental health	67.8 (3.9)	68.8 (4.1)
Role difficulties	66.4 (5.8)	73.7 (4.9)
Dependency	63.2 (4.8)	62.3 (4.7)
Driving	45.5 (13.8)	52.8 (14.7)
Color vision	68.1 (7.8)	59.2 (8.6)
Peripheral vision	73.7 (6.8)	63.2 (7.7)
**Composite**	**68.8 (3.1)**	**70.4 (3.2)**

Supplementary Table S4. Vision-related quality of life by the NEIVFQ-25.

	Duration of Dantrolene Treatment (months)	
	**0**	**6**
**Scale - Mean (SEM)**	(n=8)	(n=8)
Physical health	77.7 (6.8)	80.4 (4.9)
Psychosocial health	65.6 (9.7)	63.3 (8.1)
**Total score**	**69.8 (8.6)**	**69.3 (6.6)**

Supplementary Table S5. Pediatric Quality of Life (PedsQL) questionnaire

SF-36v Score

**Duration (months)**	**0**	**6**	***p*-value**
Count	11	12	
Mean (SEM)	115.4 (2.5)	108.8 (2.7)	0.3

Supplementary Table S6. Physical and mental health metrics as assessed by the SF-36v.

Duration (months)		0		6			0		6		
Cytokine	Norml range	Median (IQR)		Median (IQR)		*p*-value	Median (IQR)		Median (IQR)		*p*-value
Fractalkine (pg/mL)	330 ± 77.6	164.91	(147.00 - 209.69)	162.03	(134.90 - 212.95)	0.136	160.48	(146.33 - 178.50)	153.92	(133.70 - 166.06)	0.091
GM-CSF (pg/mL)	16.8 ± 5.1	12.88	(7.79 - 30.11)	11.81	(6.62 - 26.48)	0.124	12.25	(6.59 - 15.20)	11.32	(6.04 - 12.14)	0.004
IFNγ (pg/mL)	10.8 ± 3.5	48.44	(39.94 - 61.60)	45.61	(36.97 - 53.84)	0.124	48	(37.25 - 51.69)	40.58	(34.93 - 46.97)	0.021
Isoprostane (ng/mL)	0.035 ± 0.006	0.191	(0.16 - 0.23)	0.189	(0.14 - 0.28)	0.118	0.196	(0.18 - 0.31)	0.189	(0.15 - 0.31)	0.041
ITAC (pg/mL)	30.8 ± 8.5	29.82	(23.30 - 38.20)	33.79	(28.23 - 46.26)	0.266	24.49	(21.32 - 32.14)	33.5	(20.19 - 43.05)	0.155
MIP-1α (pg/mL)	29.4 ± 3.9	8.9	(0.73 - 14.90)	9.03	(0.08 - 13.14)	0.975	9.2	(3.57 - 14.77)	10.7	(2.34 - 13.21)	0.859
MIP-1β (pg/mL)	8.4 ± 3.3	14.13	(9.56 - 17.90)	20.75	(17.02 - 27.93)	0.017	14.84	(10.39 - 18.31)	23.61	(20.35 - 28.41)	0.013
MIP-3α (pg/mL)	15.2 ± 9.1	19.88	(18.73 - 25.33)	19.96	(16.23 - 24.01)	0.076	19.26	(16.89 - 21.09)	17.7	(15.80 - 19.98)	0.091
TNFα (pg/mL)	5.9 ± 2.3	6.3	(5.25 - 7.88)	6.81	(5.50 - 7.99)	0.463	5.67	(5.13 - 9.90)	6.81	(6.01 - 8.81)	0.374
IL-1β (pg/mL)	1.1 ± 0.4	1.59	(1.12 - 2.06)	1.34	(1.12 - 1.73)	0.019	1.55	(1.01 - 2.02)	1.34	(0.88 - 1.57)	0.010
IL-2 (pg/mL)	4.7 ± 2.0	2.92	(2.14 - 3.68)	2.81	(1.76 - 3.25)	0.039	2.63	(1.67 - 3.55)	2.17	(1.58 - 2.85)	0.013
IL-4 (pg/mL)	61.7 ± 16.2	35.83	(20.75 - 331.40)	29.82	(14.10 - 150.49)	0.163	61.11	(19.24 - 313.97)	42.66	(13.70 - 270.50)	0.594
IL-5 (pg/mL)	5.2 ± 1.6	3.77	(2.33 - 7.61)	3.1	(1.82 - 5.32)	0.163	2.63	(1.67 - 8.93)	2.39	(1.36 - 7.77)	0.534
IL-6 (pg/mL)	7.3 ± 2.6	3.67	(2.35 - 28.36)	3.01	(1.86 - 19.67)	0.266	8.72	(2.90 - 28.53)	10.18	(2.44 - 26.44)	0.859
IL-7 (pg/mL)	18.0 ± 5.5	17.86	(15.17 - 21.61)	17.42	(15.98 - 20.41)	0.723	16.68	(14.08 - 19.21)	17.39	(15.83 - 18.51)	0.929
IL-8 (pg/mL)	10.4 ± 5.6	8.82	(5.52 - 56.49)	8.91	(7.13 - 52.88)	0.435	11.24	(6.13 - 60.59)	12.16	(7.46 - 56.59)	0.929
IL-10 (pg/mL)	20.4 ± 10.8	13.54	(9.71 - 24.43)	12.45	(8.20 - 21.53)	0.177	18.04	(12.05 - 26.43)	12.53	(8.45 - 21.80)	0.075
IL-12P70 (pg/mL)	4.1 ± 1.8	3.95	(3.23 - 4.33)	3.83	(3.06 - 4.25)	0.435	3.57	(2.73 - 4.23)	3.19	(2.70 - 4.04)	0.248
IL-13 (pg/mL)	10.3 ± 5.3	8.3	(6.70 - 30.21)	7.44	(4.30 - 17.33)	0.015	8.68	(6.37 - 32.57)	7.57	(4.98 - 27.06)	0.050
IL-17α (pg/mL)	12.1 ± 3.3	10.16	(7.08 - 12.33)	8.2	(5.53 - 9.35)	0.076	8.9	(5.40 - 10.27)	8.2	(5.10 - 8.87)	0.021
IL-21 (pg/mL)	4.9 ± 2.9	5.4	(3.67 - 7.35)	4.76	(2.67 - 6.09)	0.039	5.82	(2.65 - 6.68)	4.87	(2.50 - 5.57)	0.008
IL-23 (pg/mL)	356 ± 290	301.49	(168.62 - 548.89)	195.95	(139.38 - 432.42)	0.084	301.49	(125.60 - 525.79)	195.95	(115.20 - 371.68)	0.131

Duration (months)	0		6			0		6			0		6		
Cytokine	Median (IQR)		Median (IQR)		*p*-value	Median (IQR)		Median (IQR)		*p*-value	Median (IQR)		Median (IQR)		*p*-value
Fractalkine (pg/mL)	229.47	(175.91 - 267.22)	207.965	(172.84 - 234.26)	0.753	164.225	(146.67 - 209.09)	160	(134.30 - 198.24)	0.239	169.5	(160.48 - 250.05)	170.09	(141.45 - 222.60)	0.345
GM-CSF (pg/mL)	33.64	(23.17 - 45.43)	28.53	(25.42 - 39.24)	0.917	15.2	(6.31 - 24.94)	12.135	(5.35 - 25.42)	0.099	12.59	(12.25 - 30.11)	11.78	(11.32 - 30.58)	0.686
IFNγ (pg/mL)	58.915	(47.28 - 83.28)	51.35	(46.42 - 71.10)	0.917	46.485	(39.34 - 58.06)	40.8	(36.06 - 52.21)	0.136	51.69	(48.00 - 61.60)	47.24	(46.70 - 53.84)	0.500
Isoprostane (ng/mL)	0.167	(0.14 - 0.19)	0.1915	(0.14 - 0.26)	0.917	0.185	(0.15 - 0.21)	0.153	(0.13 - 0.28)	0.221	0.213	(0.19 - 0.27)	0.2055	(0.20 - 0.25)	0.273
ITAC (pg/mL)	40.145	(31.92 - 58.93)	38.82	(31.36 - 51.36)	0.917	32.135	(27.59 - 39.17)	36.815	(30.12 - 47.17)	0.480	22.1	(20.53 - 23.99)	30.55	(18.24 - 35.94)	0.345
MIP-1α (pg/mL)	5.755	(0.71 - 13.40)	3.86	(0.08 - 8.68)	0.893	8.745	(0.57 - 15.34)	8.335	(0.08 - 11.34)	0.799	8.9	(6.40 - 12.86)	12.14	(0.08 - 13.14)	0.715
MIP-1β (pg/mL)	10.74	(6.37 - 14.79)	10.955	(5.73 - 16.05)	0.753	11.72	(9.43 - 15.53)	20.35	(15.68 - 28.11)	0.034	18.72	(15.57 - 19.25)	21.85	(17.51 - 23.87)	0.345
MIP-3α (pg/mL)	24.315	(21.55 - 30.86)	23.075	(21.17 - 25.14)	0.463	20.495	(18.81 - 25.44)	19.98	(16.86 - 22.98)	0.136	19.26	(18.73 - 22.88)	17.7	(16.21 - 24.01)	0.225
TNFα (pg/mL)	7.025	(6.44 - 7.52)	6.275	(5.24 - 7.52)	0.917	7.255	(6.03 - 9.74)	7.62	(6.06 - 10.43)	0.937	4.8	(4.72 - 5.00)	5.33	(5.15 - 6.81)	0.138
IL-1β (pg/mL)	1.71	(1.27 - 3.33)	1.43	(1.15 - 2.58)	0.463	1.445	(1.11 - 2.04)	1.36	(1.07 - 1.68)	0.117	1.72	(1.55 - 2.06)	1.2	(1.13 - 1.99)	0.043
IL-2 (pg/mL)	3.405	(2.79 - 4.97)	3.03	(2.53 - 4.83)	0.917	2.655	(2.06 - 3.69)	2.49	(1.74 - 2.95)	0.099	3.54	(2.92 - 3.68)	2.85	(2.43 - 3.32)	0.225
IL-4 (pg/mL)	32.715	(28.95 - 257.51)	26.455	(22.34 - 33.68)	0.116	32.715	(26.16 - 120.62)	29.41	(20.09 - 96.52)	0.388	331.4	(20.75 - 1038.08)	29.82	(13.76 - 1058.54)	0.225
IL-5 (pg/mL)	4.465	(3.80 - 6.97)	4.09	(3.33 - 4.41)	0.173	3.59	(2.55 - 5.50)	3.07	(2.20 - 4.71)	0.530	7.61	(2.13 - 16.08)	4.03	(0.93 - 17.02)	0.138
IL-6 (pg/mL)	2.565	(2.16 - 22.01)	1.865	(1.38 - 2.37)	0.116	3.4	(2.31 - 18.65)	3.15	(1.87 - 13.46)	0.530	28.36	(2.66 - 107.27)	1.87	(1.80 - 113.14)	0.225
IL-7 (pg/mL)	20.495	(19.31 - 27.58)	20.08	(16.81 - 23.73)	0.463	16.995	(14.96 - 21.67)	17.48	(15.91 - 19.92)	0.875	19.29	(17.86 - 20.56)	17.39	(16.98 - 20.41)	0.345
IL-8 (pg/mL)	6.71	(4.94 - 28.44)	7.03	(5.38 - 8.54)	0.345	8.36	(5.89 - 29.67)	9.55	(7.18 - 23.78)	0.937	35.28	(5.49 - 221.21)	7.44	(7.13 - 200.53)	0.225
IL-10 (pg/mL)	10.13	(7.38 - 15.72)	9.145	(7.40 - 15.53)	0.917	15.435	(9.21 - 25.43)	14.91	(8.58 - 21.67)	0.347	13.54	(11.27 - 17.45)	8.28	(8.15 - 12.53)	0.345
IL-12P70 (pg/mL)	4.18	(4.01 - 4.49)	4.155	(3.91 - 4.32)	0.753	3.825	(3.25 - 4.40)	3.52	(2.98 - 4.40)	0.530	4.17	(3.23 - 4.19)	3.83	(3.65 - 4.21)	0.686
IL-13 (pg/mL)	7.62	(6.90 - 24.70)	6.22	(4.52 - 7.39)	0.116	8.235	(6.81 - 15.13)	7.45	(6.11 - 10.30)	0.117	30.21	(6.03 - 70.99)	4.3	(3.46 - 58.20)	0.043
IL-17α (pg/mL)	11.585	(8.73 - 12.41)	8.685	(6.98 - 11.71)	0.753	9.54	(6.61 - 11.21)	8.11	(5.48 - 9.18)	0.084	10.16	(8.05 - 12.44)	9.07	(6.21 - 9.35)	0.500
IL-21 (pg/mL)	4.855	(4.18 - 8.37)	4.205	(3.22 - 6.11)	0.753	5.065	(3.42 - 7.85)	4.815	(2.58 - 6.21)	0.050	5.4	(4.04 - 6.00)	3.08	(3.08 - 5.05)	0.500
IL-23 (pg/mL)	408.225	(247.06 - 596.50)	276.86	(144.92 - 557.31)	0.345	270.86	(160.13 - 659.13)	198.54	(147.87 - 469.44)	0.308	512.19	(267.56 - 539.38)	195.95	(134.18 - 432.42)	0.043

Supplemental Table 7. Levels of inflammatory cytokines and isoprostane
